# Cadmium Ecotoxic Effects on Embryonic *Dmrt1* and *Aromatase* Expression in *Chrysemys picta* Turtles May Implicate Changes in DNA Methylation

**DOI:** 10.3390/genes13081318

**Published:** 2022-07-24

**Authors:** Beatriz Mizoguchi, Nicholas E. Topping, Andrew M. Lavin, Nicole Valenzuela

**Affiliations:** Department of Ecology, Evolution and Organismal Biology, Iowa State University, Ames, IA 50011, USA; topping@iastate.edu (N.E.T.); andrewlav208@gmail.com (A.M.L.); nvalenzu@iastate.edu (N.V.)

**Keywords:** sex determination, epigenetic modification by DNA methylation, cadmium, sexual testis and ovary development, ecotoxicology of endocrine, transcriptional, developmental disruptors, reptile vertebrates

## Abstract

Temperature-dependent sex determination (TSD) decides the sex fate of an individual based on incubation temperature. However, other environmental factors, such as pollutants, could derail TSD sexual development. Cadmium is one such contaminant of soils and water bodies known to affect DNA methylation, an epigenetic DNA modification with a key role in sexual development of TSD vertebrate embryos. Yet, whether cadmium alters DNA methylation of genes underlying gonadal formation in turtles remains unknown. Here, we investigated the effects of cadmium on the expression of two gene regulators of TSD in the painted turtle, *Chrysemys picta*, incubated at male-producing and female-producing temperatures using qPCR. Results revealed that cadmium alters transcription of *Dmrt1* and *aromatase*, overriding the normal thermal effects during embryogenesis, which could potentially disrupt the sexual development of TSD turtles. Results from a preliminary DNA methylation-sensitive PCR assay implicate changes in DNA methylation of *Dmrt1* as a potential cause that requires further testing (*aromatase* methylation assays were precluded).

## 1. Introduction

Concerns over pollution and contamination of water bodies and soil have emerged in the past decades, and attention is growing due to the potential harm they can cause to living organisms. For instance, disruption of sex ratios in natural populations of vertebrates by such contaminants can cause population decline or extinction [1,2]. This occurs because some pollutants can alter sex determination, the biological process that sets the sexual fate of developing embryos and which ranges between two ends of a continuum [3,4,5]. On one end is genotypic sex determination (GSD), in which a genetic component triggers the molecular and hormonal cascade that commits the gonad to become a testis or an ovary. On the other end is environmental sex determination (ESD), in which an environmental factor (such as temperature—TSD, pH, population density, among others) is responsible for initiating the sexual development cascade [3,4,6]. In TSD, the incubation temperature experienced during a specific embryonic development period is the triggering factor [7]. TSD is predominant among turtle species, while GSD in the form of sex chromosomes evolved independently multiple times [8]. The painted turtle (*Chrysemys picta*) is a TSD species lacking sex chromosomes [9].

Sex determination in *C. picta* may be mediated in part by DNA methylation [10], an epigenetic modification that alters gene expression patterns without changing the DNA sequence, by substituting the 5′carbon of a cytosine with a methyl group. Depending on its location and context, DNA methylation may repress, activate, or modulate gene expression [11]. More often, methylated cytosines physically block access of the transcription machinery to the DNA [12], silencing methylated genes. In general, DNA methylation is an important molecular mechanism that organisms use to respond to environmental stimuli [13,14], and it is critical for sex determination in turtles and other reptiles with TSD [15,16,17,18]. For instance, in *Alligator mississipiensis*, the promoter region of *Sox9*, a gene involved in male sexual development, is methylated at female-producing temperatures, repressing its expression in developing ovaries [15]. Another example of a male development gene regulated by methylation is *Dmrt1*, which exhibits hypermethylation (and thus is downregulated) in developing females compared to males in TSD and GSD fish [19,20,21,22]. Importantly, *Dmrt1* is under additional epigenetic control via histone methylation in the TSD turtle *Trachemys scripta*, where the histone demethylase KDM6B removes trimethylation from H3K27, a histone close to the *Dmrt1* promoter [23]. Third, *aromatase*, a key gene for female development, is regulated in *T. scripta* by DNA methylation at specific CpG sites on its promoter region. Specifically, CpG sites located close to binding sites for *Foxl2*, a known *aromatase* regulator, and close to the TATA box, an RNA-polymerase recruiter site, are hypomethylated at female-producing compared to male-producing temperatures [24]. Furthermore, recent studies demonstrated a global sexually dimorphic DNA methylation during gonadogenesis of *Lepidochelys olivacea* sea turtles [17], a pattern observed in gonads of *C. picta* hatchlings incubated in different temperatures, including at gene regulators of sexual development [10]. Yet, how contaminants such as cadmium might alter DNA methylation of these important genes during embryogenesis in developing male and female turtles remains unknown.

Contaminants present in water or soil during the incubation period may affect sex determination, mainly by disrupting the hormonal balance during sexual development [1] or altering DNA methylation of relevant sexual development genes. For instance, cadmium that is present in multiple industrial processes [25] can disrupt DNA methylation patterns and potentially affect gonadal development [26,27]. Here we investigated the effects of cadmium exposure on DNA methylation of *C. picta* turtles during three different embryonic stages and the consequent effects on gene expression patterns of a gene important for testicular formation (*Dmrt1*) and a key regulator of ovarian formation (*aromatase*). In the absence of cadmium effects, we expect *Dmrt1* to be hypermethylated (and thus downregulated) in developing females (at 31 °C) compared to males (at 26 °C), and *aromatase* to be hypermethylated (and thus downregulated) in developing males (at 26 °C) compared to females (at 31 °C), opposite transcriptional patterns to those observed typically at those temperatures in *C. picta* embryos [28,29].

## 2. Materials and Methods

### 2.1. Egg Collection, Incubation, and Cadmium (Cd) Treatment

Freshly laid eggs were collected from a turtle farm in Iowa and transported in moist vermiculite to the laboratory for incubation following our standard protocols [30], with some modifications. Briefly, eggs were cleaned, marked with unique IDs, and randomly assigned to incubation boxes with moist sand. Before the onset of incubation, 1 mL of a solution of 1 μg/mL of CdCl_2_ diluted in water was applied topically to each egg. This dose was chosen because previous studies at female-producing temperature using ecologically relevant cadmium doses found that 1 μg/g egg reduced germ cells in neonate gonads of *Trachemys scripta* turtles, whereas the observed germ cell reduction using 0.1 μg/g was not statistically significant, but importantly, that *C. picta* appears much more susceptible to cadmium than *T. scripta* [27]. Thus, given a *C. picta* egg mass of ~6 g [31,32,33], a dose of 1 μg per egg (~0.17 μg/g egg) fell within the ecologically relevant range [27]. Water was chosen as solvent because CdCl_2_ is hygroscopic and highly soluble in water, the turtle eggshell absorbs water and water-soluble contaminants readily [34,35], and to avoid unknown effects of other solvents. Eggs were incubated at 26 °C (male-producing temperature, MPT) (*n* = 80) and 31 °C (female-producing temperature—FPT) (*n* = 88), of which 40 were intended for hatching to test cadmium effects on sex ratios and the remainder were intended for assessment of embryonic gene expression and DNA methylation. The position of the boxes inside the incubator was rotated daily between left/right side and top/bottom shelfs to account for any thermal gradient that might exist, following standard procedures [30,36]. Moisture was maintained by replacing evaporated water (determined by weight reduction of the box) weekly. Embryonic development and viability were assessed by egg candling weekly. Embryos and tissues were dissected at Yntema’s stages 09 (before the thermosensitive period—TSP), 15 (just prior to the onset of TSP), and 22 (end of the TSP), and stored in RNA later (Invitrogen) at −20 °C until processing. Because the gonadal primordium cannot be separated in stage 9 embryos, trunks were sampled at that early stage. Adrenal-kidney-gonad (AKG) complexes were sampled at stage 15 because the gonads could not be separated from the AK. Stage 15 gonads are bipotential in *Chrysemys* [37]. Lastly, separated gonads were sampled from stage 22 embryos. While some of the differences among stages are likely attributable to differences in the tissues sampled, our approach permits testing for differences in gene expression and methylation between FPT and MPT at each developmental period, as well as between embryos exposed to cadmium (this study) and embryos incubated in the absence of cadmium under otherwise identical conditions, which serve as negative controls. Hatchlings obtained after 45 days of incubation were kept in water tanks at 25 °C and fed commercial turtle food for three months before autopsy to ensure gonadal differentiation was complete before sexing by gonadal inspection. All procedures were done in accordance with guidelines and regulations approved by the IACUC of Iowa State University.

### 2.2. DNA and RNA Extractions, cDNA Conversion

DNA and RNA were extracted from embryo trunks (stage 09, *n* = 15 per temperature), AKG complexes (stage 15, *n* = 12 per temperature), and gonads (stage 22, *n* = 10 per temperature) using Qiagen AllPrep DNA/RNA ™ Mini (stages 9 and 15) and Micro (stage 22) kits, following the manufacturer’s instructions. Differences in the sample size per stage were due to embryonic mortality at later stages of development due to cadmium exposure. RNA and DNA were quantified using a NanoDrop Spectrophotometer. DNA was stored at −20 °C until further use.

The RNA extraction protocol from the extraction kits included a treatment with DNAse to reduce gDNA. RNA quality was assessed by the presence of ribosomal bands in 1% agarose gels. RNA was stored at −80 °C until 500 ng of RNA was converted to cDNA using Invitrogen SuperScript™ VILO™ Synthesis kit following the manufacturer’s instructions; cDNA was stored at −20 °C until further use.

### 2.3. qPCR Assay and Data Analysis

To assess whether cadmium exposure altered gene expression patterns of *Dmrt1*, we carried out qPCR reactions in an Mx3000P real-time PCR thermal cycler (Stratagene), using the cDNA produced as described above. For *Dmrt1*, we used a TaqMan probe-based multiplex qPCR to amplify a fragment of only the canonical isoform of this gene (147 bp) and a fragment of the *ß-actin* transcript as a reference gene (138 bp), following previous *Dmrt1* profiling in this species [38]. All primers used in this study are listed in Table 1. We used the IDT Prime Time Gene Expression Master Mix, which contains the DNA polymerase, dNTPs, MgCl_2_, enhancers, and stabilizers, in concentrations undisclosed by the manufacturer. Multiplex optimization reactions with primers concentration of 500 nM and probes concentration of 500 nM (*Dmrt1*) and 250 nM (*ß-actin*) were carried out, with 5 μL of commercial master mix, *ß-actin* and *Dmrt1* forward and reverse primers, *ß-actin* and *Dmrt1* Taqman probe, 2 μL of cDNA (50 ng), and water up to 15 μL.

For *aromatase*, a gene with no known alternative spliceoforms, we carried out SYBR Green reactions to amplify a 168 bp transcript fragment of *aromatase*, using *GAPDH* as a reference gene (127 bp amplicon), following [39,40,41]. Reactions were prepared using Brilliant^®^ SYBR^®^ Green qPCR Master Mix with *aromatase* and *GAPDH* primers in separate qPCR reactions.

For all genes, qPCR conditions included an initial denaturing step at 95 °C for 3 min followed by 50 cycles of 95 °C for 30 s and 60 °C for 1 min. All reactions were run in duplicate. We generated standard curves by pooling cDNA from all samples (100 ng per individual) and then serially diluted the pooled cDNA using a 1:5 ratio to obtain a total of eight standards. Standards were included in duplicate in each qPCR plate to generate standard curves to calculate qPCR efficiency and R^2^ values.

All samples showed a coefficient of variation <10% between technical replicates; thus, they were all included in the analysis. *ß-actin* and *GAPDH* displayed constant expression across stages and temperatures in our study. We performed a Student’s *t*-test in R [42] to test for differences in the normalized ratio of *Dmrt1* to *ß-actin*, and of *aromatase* to *GAPDH*, between temperatures at each stage and assessed significance at an α of 0.05. As negative controls, we leveraged previous qPCR profiling of *Dmrt1* and *aromatase* expression of stage 9, 15, and 22 *C. picta* in embryos incubated at identical thermal conditions but in the absence of cadmium exposure as reported elsewhere [38].

### 2.4. PCR Test of DNA Methylation

**Figure 1:**
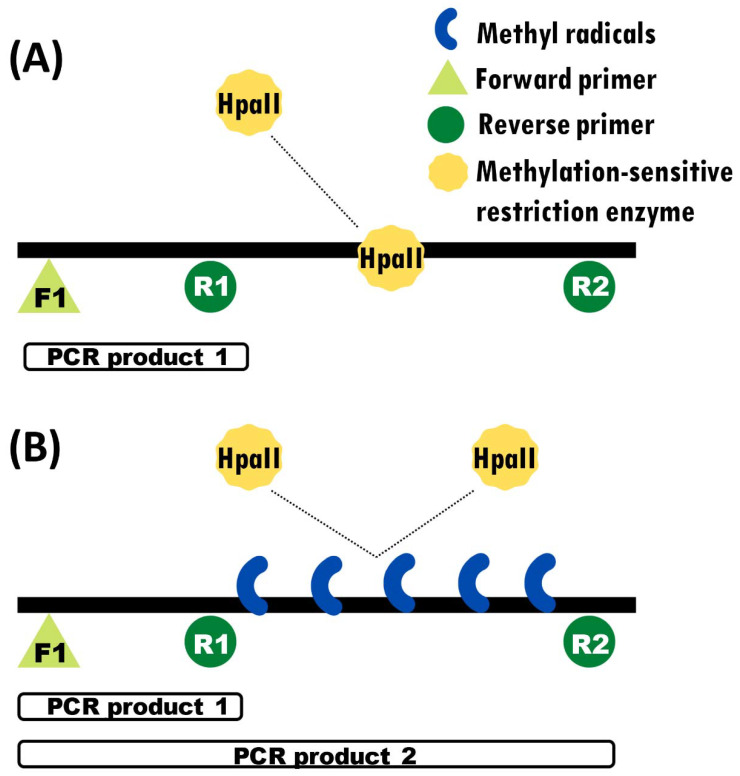
Experimental design of the methylation-sensitive PCR assay. Black bars denote the target DNA sequence, onto which the relative position of the primers, restriction sites, and methylation marks are illustrated. White bars denote the relative size of fragments amplified by PCR. (**A**) Amplification of only one PCR product due to the DNA digestion by HpaII. (**B**) Amplification of two PCR products thanks to the protection conferred by the DNA methyl groups, which prevents DNA digestion by the restriction enzyme.

In order to assess DNA methylation status, we first digested the DNA with a DNA-methylation-sensitive restriction enzyme, then amplified digested and undigested DNA via PCR using a combination of primers designed specifically to produce differential amplification if the DNA is methylated (and thus digested) or not [43]. We used this method in previous studies of DNA methylation in *C. picta* hatchlings (not embryos) for the gene *Fezf2* [10]. This primer design included one forward primer (F1) and two reverse primers (R1 and R2), one of which (R1) binds closer to F1, and one (R2) which binds further away from F1 (Figure 1). Primers F1 + R1 amplify a smaller region nested within the larger region amplified by F1 + R2. Primers F1 and R1 flank a non-methylated area, which should be amplified irrespective of DNA methylation status and thus serve as a positive control. In contrast, primers F1 and R2 flank a region that encompasses the differentially methylated CpG island and amplify depending on the methylation status of this area. Methylated regions are protected from the restriction enzyme and thus are not digested, resulting in two amplicon bands in the gel electrophoresis, while unmethylated regions are digested and result in a single PCR amplicon band. We designed primers at the promoter regions of *Dmrt1*, focusing on sequences that contained the restriction site of HpaII (CCGG).

Before PCR amplification, the DNA from embryos of the three stages examined was digested with HpaII (Thermo Fisher, Waltham, MA, USA) following the manufacturer’s instructions. Primers for *aromatase* and *Dmrt1* were designed at their promoter regions using the design described above. PCR was carried out separately using DNA subjected to the restriction enzyme and undigested (control) samples in 15 μL reactions with 1× Taq buffer, 1.5 mM MgCl_2_, 0.2 mM dNTPs, 0.4 μM of each primer (F1, R1 and R2), 0.4 U Taq polymerase, and 10.5 μL water, with 50 ng of DNA as template. PCR conditions included an initial denaturing step at 94 °C for 3 min, followed by 35 cycles of denaturing at 94 °C for 30 s, annealing at 58 °C for 30 s, and extension at 72 °C for 1 min. PCR products were visualized in a 1% agarose gel stained with EtBr, and their size was estimated by a 1 kb plus ladder (Invitrogen). DNA methylation status was determined qualitatively by the intensity of the amplicon bands in the agarose gel.

In the case of genes that are hypermethylated in males, we expected to see a single band in females (corresponding to the small amplicon produced by F1 + R1 primers in the nested non-methylated region) and two bands in males (the common smaller band from F1 + R1 primers and a larger band from F1 + R2 primers). The opposite is expected in cases when genes are hypermethylated in females: one band in males and two bands in females. Because the DNA-methylation-sensitive PCR assay was not conducted during the previous study [38], our experimental design for the methylation assessment lacks a direct negative control with embryos not exposed to cadmium, such that our PCR results are preliminary and should be taken as suggestive until further testing, and interpreted only in the context of the gene expression profiling.

## 3. Results

### 3.1. Cadmium Induces High Embryonic Mortality

Only 6.67% of the 40 eggs incubated until hatching to assess sex ratios when exposed to cadmium survived. While the mortality observed in parallel incubation experiments conducted that same year was particularly high (60–65%) for unknown reasons [44], the mortality of cadmium-treated embryos exceeded that baseline by >30%. Early embryo mortality under cadmium exposure (before stage 09 of development) affected 24% of the eggs (*n* = 11), while 49% of the eggs (*n* = 22) died during late development (between stages 23 and 26), and 8% (*n* = 4) of the eggs died after they pipped or hatched. Consequently, only three eggs hatched and survived to three months of age. These three hatchlings were not sex-reversed (*n* = 2 males from 26 °C and *n* = 1 female from 31 °C). Other than the increased embryonic mortality, no obvious effect of cadmium exposure was observed in the morphology and anatomy of the three surviving hatchlings. However, the small number of surviving individuals precluded testing for the effect of cadmium on sex reversal as originally intended, such that further studies are warranted using alternative cadmium doses. Of the eggs incubated for embryonic methylation and gene expression analysis, 15, 12, and 10 embryos were collected from each temperature at stages 9, 15, and 22, respectively.

### 3.2. Cadmium Exposure Alters Gene Expression

**Figure 2:**
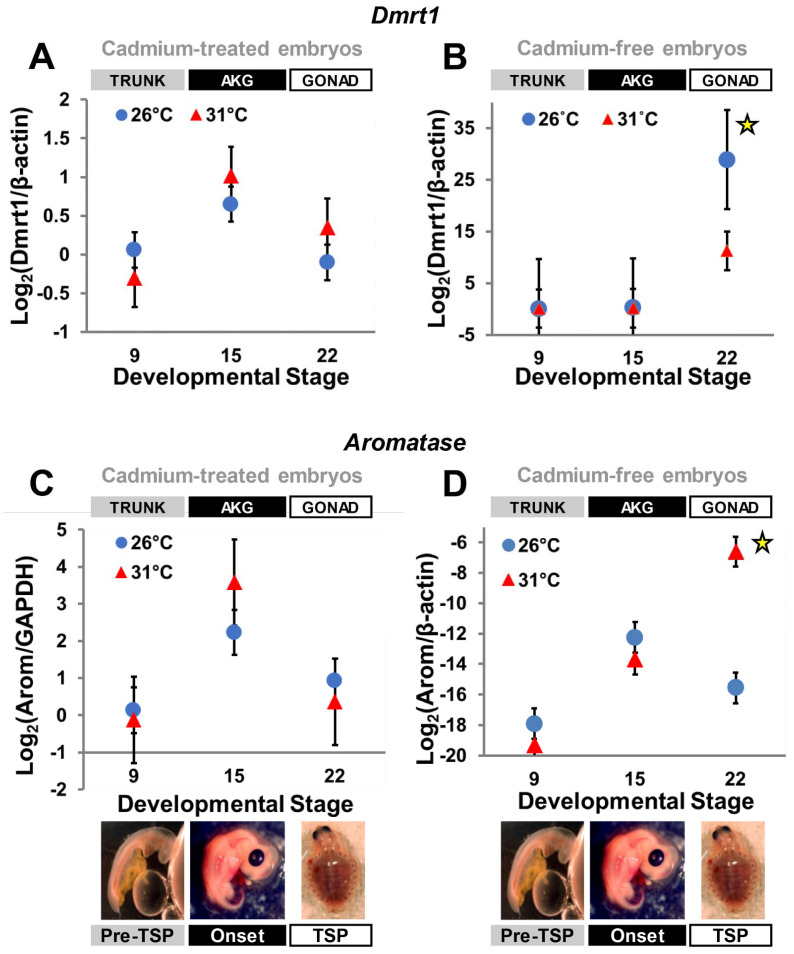
*Dmrt1* and *aromatase* transcription under and without cadmium exposure. Data derived from this study (**A**,**C**), and from Mizoguchi and Valenzuela 2020 [38] (**B**) and Valenzuela et al. 2013 [28] (**D**), which serve as negative control. Bars represent standard deviations. In embryos exposed to cadmium (**A**,**C**), differences in expression between temperatures were not significant at any of the stages examined, whereas in the absence of cadmium exposure, *Dmrt1* (**B**) and *aromatase* (**D**) are normally upregulated at stage 22 at MPT or FPT, respectively (indicated by the yellow star symbol).

All standard curves for the qPCR reactions exhibited a R^2^ > 0.98, and negative controls showed no amplification. Our quantitative results indicate that both *Dmrt1* and *aromatase* transcriptional patterns were altered by exposure to cadmium. Specifically, *Dmrt1* transcription was higher, but not significantly so (Table 2), in presumptive developing females (i.e., at FPT—31 °C) at stages 15 (TSP-onset) and 22 (late-TSP), rather than being upregulated in presumptive males (i.e., at MPT—26 °C) at stage 22 as occurs under uncontaminated conditions (Figure 2A,B). *Aromatase* transcription at MPT was also affected, as expression peaked at stage 15 and dropped at stage 22, with no significant upregulation (Table 2) at FPT by stage 22, as occurs under normal conditions [28,29] (Figure 2C,D). Thus, cadmium exposure eliminated the differential transcription characteristic of *Dmrt1* and *aromatase* expression (*p* > 0.05 in all cases) as expected during typical development [28,29,38].

### 3.3. Cadmium Exposure Induces Similar DNA Methylation Profiles of Dmrt1, a Testicular Development Regulator

**Figure 3:**
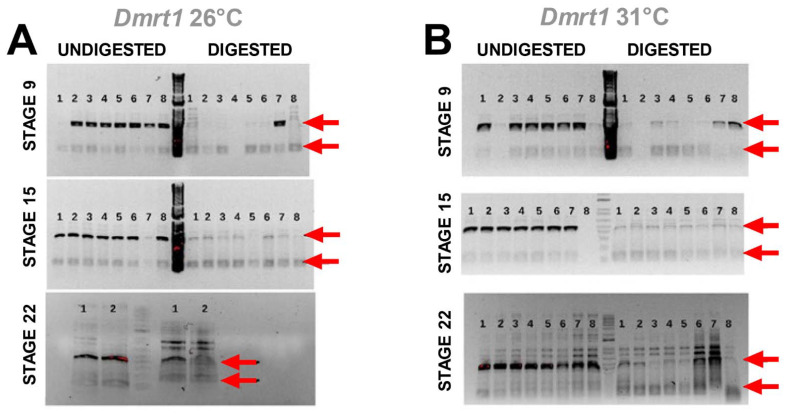
Agarose gels of DNA methylation-sensitive PCR assay for *Dmrt1* at MPT (**A**) and FPT (**B**). Invitrogen’s 1 kb Plus DNA ladder was used. Arrows show the size of the two amplicons expected (120 bp and 438 bp) when DNA is methylated and thus protected from digestion by the restriction enzyme, as detailed in the text.

Results from the methylation-sensitive PCR assay showed similar levels of DNA methylation between temperatures for *Dmrt1* at all stages (Figure 3). This was assessed qualitatively by the reduction of PCR amplification (fainter bands) of the larger amplicon in the digested DNA samples compared to the undigested controls to a similar level at both incubation temperatures, indicating that DNA was equally unprotected at 26 °C and 31 °C from digestion by DNA methylation. This similarity between MPT and FPT reflects either demethylation or inhibition of DNA methylation due to cadmium exposure, which should lead to similar levels of transcription in developing males and females. Unfortunately, a freezer malfunction ruined the samples before PCR amplification of *aromatase*, precluding *aromatase* methylation tests.

## 4. Discussion

In this study, we investigated for the first time the effects of cadmium exposure on the expression of two gene members of the sexual development network, *Dmrt1* and *aromatase* (Figure 2). *Dmrt1* encodes a transcription factor involved in male-specific differentiation. *Aromatase* is a cytochrome P450 enzyme responsible for aromatizing testosterone into estrogen and is key for ovarian formation [24,33]. We observed that cadmium exposure during embryogenesis alters gene expression patterns in ways consistent with a potential to disrupt sexual development.

Cadmium is known to affect DNA methylation by interacting with the DNA-binding domain of DNA methyltransferase, the enzyme responsible for establishing DNA methylation marks, leading to hypomethylation [26]. Concordantly, we observed quantitative changes in gene expression that match this mode of action for cadmium. For instance, cadmium exposure can alter gene expression when gene promoters are regulated by DNA methylation, as is the case for *Dmrt1* in fish [13,20,21,22], *aromatase* in TSD turtles and fish [19,24], and *Sox9* in alligators [15]. These genes are differentially transcribed in the developing embryo of *C. picta* turtles [28,29,38]. Yet, cadmium obliterated the significant differential expression of *Dmrt1* and *aromatase* typically observed between MPT and FPT in this species (Figure 2). Indeed, no significant differential expression was detected at any stage examined here, whereas in previous studies, *Dmrt1* and *aromatase* were significantly upregulated at stages 19 and 22 at MPT (*Dmrt1*) or FPT (*aromatase*), respectively [30,31,34]. This is a notable finding, given that such abnormal expression patterns could greatly affect downstream genetic pathways and, consequently, sexual development. Furthermore, the alteration of the transcription of both a male- and a female-inducing gene during our experiment indicates that cadmium can interfere in sexual development by directly altering gene expression patterns of relevant sex-related genes. Previous studies demonstrated that cadmium can affect germ cell abundance in ovaries of neonatal TSD turtles, but no sex reversal was reported [27]. Whether cadmium induces sex reversal remains unknown, particularly at MPT, because high mortality precluded sex ratio analyses here, and the previous work only tested FPTs [27].

For instance, upregulation of *aromatase* at MPT by any factor could lead to higher estrogen production, which could directly interfere with the male sexual development pathway. Indeed, estrogen affects the expression of steroidogenic factor-1 (*Sf-1*, *NR5A1*), an activator of the anti-Mullerian hormone (AMH) [45], critical for testis development. On the other hand, the downregulation of *aromatase* in females, as occurs by *aromatase* inhibitors, leads to sex reversal or masculinization of gonads [36,37]. Further, *Dmrt1* expression responds rapidly to *aromatase* inhibition [18], revealing *Dmrt1*’s early role in initiating male differentiation in some TSD turtles. Although we did not observe any sex reversal in our study due to the increased mortality induced by cadmium, previous studies in *C. picta* observed oocyte apoptosis in *C. picta* neonates after cadmium exposure, perhaps due to the disruption of *aromatase* expression [27]. Similarly, *aromatase* inhibition was linked to oocyte apoptosis in juvenile and adult zebrafish [38,39]. Furthermore, downregulation of *Dmrt1* at MPT induces the upregulation of *Foxl2*, an important gene in the female sexual development pathway, and a known inhibitor of *Sf-1* [46]. *Foxl2* also inhibits the expression of *Sox9*, another key male development gene [18]. Moreover, experimental knock-down of *Dmrt1* feminizes embryos, inducing elongated gonads and the development of oviducts or even ovotestis, whereas *Dmrt1* overexpression at FPT leads to masculinization or complete sex reversal [18].

Our transcription profiling results for *Dmrt1* (lack of significant differential expression) were associated with the qualitative patterns of DNA methylation assessed by the DNA methylation-sensitive PCR assay. Specifically, results from the PCR test are consistent with cadmium having altered DNA methylation, as they revealed a reduction of PCR amplification of the larger amplicon (this larger amplicon was expected if DNA was methylated) in the digested DNA samples compared to the undigested controls for both genes, to similar levels at both temperatures. The observation of similar methylation-sensitive PCR amplification at MPT and FPT suggests that DNA methylation levels were equivalent at both temperatures, perhaps because cadmium caused de-methylation at FPT. However, instances where chronic exposure to cadmium causes hypermethylation exist [26], such that further studies are warranted to determine the exact mode of action of cadmium in *C. picta*.

If DNA-methylation was indeed altered, and given that the transcription of both *Dmrt1* and *aromatase* was affected by cadmium exposure, a female-inducing gene that is normally upregulated at warm temperatures and a male-inducing gene that is normally upregulated at colder temperatures, we hypothesize that cadmium affects DNA methylation at a global genome level in turtles, rather than just targeting specific genes. Such effect would agree with previous studies that identified changes in global methylation in the European eel, human embryo fibroblast, and hens, associated with cadmium exposure [47,48,49]. If true for turtles, then it would be expected that other genes regulated by DNA methylation were likely affected, consistent with the increased mortality observed in our study.

In conclusion, we detected for the first time that embryos exposed to cadmium suffer increased mortality and experienced altered expression of gene regulators of sexual development. Our preliminary data suggest that this modified *Dmrt1* transcription may be associated with changes in DNA methylation, but strong inferences are precluded in the absence of methylation-sensitive PCR data from embryos not exposed to cadmium. Despite these caveats, our study contributes to the growing body of work highlighting the detrimental consequences of exposure to chemicals, especially endocrine-disrupting contaminants (EDCs), on gene expression, gonadal development, and sex reversal (reviewed in [1]). Because cadmium is a metal that accumulates in organisms, including the painted turtle, and in yolk [50], cadmium is an environmental contaminant that poses substantial dangers to sexual development [27,47,48,49]. Because no contaminant is found alone in nature, but instead in mixtures, their effects can be seen even at smaller doses [51]. Such exposure can lead to higher mortality rates, abnormal sexual development, decreased fertility, and generally lower fitness [1]. Natural populations are vulnerable to soil and water contamination, and our findings help illuminate some of the mechanism by which contaminants interfere in embryonic development. Our work should help guide broader research to aid managers in better preserving turtle populations and determining safe thresholds for human exposure to similar contaminants.

## Figures and Tables

**Table 1:** Primers and probes used in this study (see text for details).

**Primers and probes used in qPCR for gene expression assay**			
	**Forward**	**Reverse**	**Probe**
*Dmrt1*	5′ CCAACACATTCAACAAACA 3′	5′ ACTGCTGTAGTAGGTGGAGTC 3′	5′/FAM/ATCAGAGGGACGGATGCTCATTCAG 3′
*ß-actin*	5′ TGTGCTGCTTACAGAGG 3′	5′ GTACGACCAGAGGCCTA 3′	5′/CY5/GCCAACAGAGAAAAGATGACACAGATC 3′
*Aromatase*	5′ CTGTATGGGAATTGGTCC 3′	5′ TAATAATGAGTGTTTCCTCTCCACT 3′	N/A
*GAPDH*	5′ GGAGTGAGTATGACTCTTCCT 3′	5′ CAGCATCTCCCCACTTGA 3′	N/A
**Primers used in methylation-sensitive PCR assay**			
	**Forward 1**	**Reverse 1**	**Reverse 2**
*Dmrt1*	5′ TGTCTTATTTGGGTCTCATC 3′	5′ GGAAGGAGCTAATGTTTCA 3′	5′ CTTTTTACACTGACAGTCCC 3′
*Aromatase*	5′ GCTAAGCAGAAAATAACTGG 3′	5′ ATGCCTTCTTTTTCACCTC 3′	5′ ACAACTGTATGTATCAGAAGTGG 3′

**Table 2:** Results of the Student’s *t*-test statistical analysis for *Dmrt1* and *Aromatase* between temperatures at each stage examined.

	*Dmrt1*			*Aromatase*		
Developmental Stage	t	df	*p*-Value	t	df	*p*-Value
Stage 09	1.17728	14	0.098	1.6955	14	0.1121
Stage 15	1.8221	11	0.0957	0.693	11	0.5027
Stage 22	1.3729	9	0.2032	1.0124	8	0.341

## Data Availability

Quantitative data used in this study are provided in the Supplementary Information, Supplementary Table S1.

## Supplementary Materials

The following are available online at https://www.mdpi.com/article/10.3390/genes13081318/s1, Supplementary Table S1: Normalized gene expression data used in this study.
