# Supplementary material for: Cadmium Ecotoxic Effects on Embryonic Dmrt1 and Aromatase Expression in Chrysemys picta Turtles May Implicate Changes in DNA Methylation
Source: Genes (Basel). 2022 Jul 24;13(8):1318. doi: 10.3390/genes13081318 (PMC9331853; doi:10.3390/genes13081318)
Supplement: Supplementary file 1 [file genes-13-01318-s001.zip › genes-1786513-SI.pdf]

Supplementary information

Cadmium Ecotoxic Effects on Embryonic *Dmrt1* and *aromatase* Expression in *Chrysemys picta* Turtles may Implicate Changes in DNA Methylation

Beatriz Mizoguchi *, Nicholas E. Topping, Andrew M. Lavin and Nicole Valenzuela

Department of Ecology, Evolution and Organismal Biology, Iowa State University, Ames, IA 50011, USA; topping@iastate.edu (N.E.T.); andrewlav208@gmail.com (A.M.L.); nvalenzu@iastate.edu (N.V.)
* Correspondence: mizoguchibeatriz@gmail.com

This Supplementary file contains:

- Supplementary Table S1: Normalized gene expression data used in this study.

Supplementary Table S1: Normalized gene expression data used in this study

STAGE	TEMPERATURE	Log2(Aromatase/GAPDH)		STAGE	TEMPERATURE	Log2(Dmrt1/βactin)
**9**	26	0.719004532		**9**	26	1.128333152
**9**	26	1.439785323		**9**	26	1.137785578
**9**	26	1.039761835		**9**	26	0.750679458
**9**	26	1.051554626		**9**	26	1.071607414
**9**	26	1.458027371		**9**	26	0.944148975
**9**	26	1.139862166		**9**	26	1.136296534
**9**	26	1.409754147		**9**	26	1.093986252
**9**	26	0.595566711		**9**	26	0.95272863
**9**	26	0.978191628		**9**	26	0.917826684
**9**	26	1.070206792		**9**	26	1.209474205
**9**	26	1.28721907		**9**	26	1.545475571
**9**	26	1.399610552		**9**	26	0.334063532
**9**	26	0.69088359		**9**	26	1.106483006
**9**	26	1.631845326		**9**	26	0.66956533
**9**	26	0.550760495		**9**	26	1.614840226
**9**	31	1.087631427		**9**	31	1.337747414
**9**	31	1.494691215		**9**	31	0.498515001
**9**	31	0.539360541		**9**	31	0.709916769
**9**	31	0.685846659		**9**	31	1.668911512
**9**	31	0.605633565		**9**	31	1.094225417
**9**	31	1.579318102		**9**	31	0.776694023
**9**	31	0.982186425		**9**	31	1.056317679
**9**	31	0.471923062		**9**	31	0.856666969
**9**	31	1.320018		**9**	31	0.41652167
**9**	31	0.406040045		**9**	31	0.718183644
**9**	31	1.372723145		**9**	31	0.817796701
**9**	31	1.558793353		**9**	31	1.004188022
**9**	31	0.342915877		**9**	31	0.783361096
**9**	31	0.93795136		**9**	31	-0.120316446
**9**	31	0.370471929		**9**	31	0.570178887
**15**	26	1.938168375		**15**	26	1.147560366
**15**	26	2.028250212		**15**	26	1.636145711
**15**	26	32.91002175		**15**	26	1.706700554
**15**	26	2.270608389		**15**	26	1.206957881
**15**	26	3.105936645		**15**	26	1.039452276
**15**	26	1.977261615		**15**	26	1.143525898
**15**	26	2.846576235		**15**	26	1.978123427
**15**	26	1.606827521		**15**	26	1.124980852
**15**	26	1.747501401		**15**	26	1.522066805
**15**	26	1.563912541		**15**	26	2.724134287
**15**	26	2.000319315		**15**	26	1.558403598
**15**	26	2.272354877		**15**	26	2.044473511
**15**	31	1.426009965		**15**	31	2.91456419
**15**	31	1.841973134		**15**	31	1.64379965
**15**	31	155.1899604		**15**	31	1.700813719
**15**	31	1.320419334		**15**	31	2.656173907
**15**	31	-8.458832141		**15**	31	2.749149294
**15**	31	1.377824095		**15**	31	1.036280965
**15**	31	2.396795577		**15**	31	1.895581312
**15**	31	2.15145769		**15**	31	1.415243922
**15**	31	-9.245621982		**15**	31	2.571479886
**15**	31	-6.745285836		**15**	31	3.237381279
**15**	31	1.574125254		**15**	31	1.227004654
**15**	31	1.018268779		**15**	31	1.181525041
**22**	26	2.07682587		**22**	26	0.186355891
**22**	26	1.716334866		**22**	26	1.301002918
**22**	26	1.727494927		**22**	26	0.403928472
**22**	26	2.173658309		**22**	26	1.293746289
**22**	26	1.381736464		**22**	26	0.719384717
**22**	26	1.998374648		**22**	26	1.517263655
**22**	26	2.039837674		**22**	26	1.017436572
**22**	26	1.908173647		**22**	26	1.202847572
**22**	26	1.984646185		**22**	26	1.383561928
**22**	31	1.944842998		**22**	26	0.294717452
**22**	31	-3.080707695		**22**	31	0.120869061
**22**	31	1.420863987		**22**	31	1.804726044
**22**	31	1.862459783		**22**	31	2.327087735
**22**	31	2.318446894		**22**	31	1.988995604
**22**	31	2.481986642		**22**	31	1.431972253
**22**	31	1.458563737		**22**	31	0.581264718
**22**	31	1.018467547		**22**	31	1.018461747
**22**	31	2.45826473		**22**	31	1.071546175
**22**	31	1.028457274		**22**	31	1.194756727
				**22**	31	1.17164611
